# An Innervated Human Skin Equivalent that Electrically Encodes Mechanical and Thermal Stimuli

**DOI:** 10.1002/advs.77105

**Published:** 2026-08-15

**Authors:** Daniele Bellantoni, Costantino Casale, Marika Sperduti, Nevio L. Tagliamonte, Giorgia Imparato, Loredana Zollo, Paolo A. Netti

**Affiliations:** ^1^ Centro di Ricerca Interdipartimentale sui Biomateriali CRIB University of Napoli Federico II Napoli Italy; ^2^ Istituto Italiano di Tecnologia‐IIT Center for Advanced Biomaterials for Healthcare Napoli Italy; ^3^ Department of Chemical Materials and Industrial Production Engineering‐DICMAPI University of Naples Federico II Naples Italy; ^4^ Research Unit of Advanced Robotics and Human‐Centred Technologies – CREO Lab Universitá Campus Bio‐Medico di Roma Rome Italy

**Keywords:** electrophysiological wave analysis, innervated human skin equivalent, mechanoception, sensory encoding, somatosensory system, thermoception

## Abstract

Understanding how peripheral sensory circuits encode mechanical and thermal stimuli requires an experimental system that combines native tissue architecture with high‐resolution electrophysiological readouts. Existing innervated in vitro skin models typically rely on chemical stimulation and optical measurements, limiting their ability to interrogate sensory encoding dynamics. Here, we present an innervated full‐thickness human skin equivalent that electrically encodes both mechanical and thermal stimuli. The construct integrates a differentiated epidermis and dermis with sensory neurons and Schwann cells forming free nerve ending‐like structures, from which the recorded activity originates, and is interfaced with high‐density microelectrode arrays. Controlled mechanical indentation and localized thermal stimulation evoke consistent, stimulus‐dependent firing patterns whose waveform morphology and temporal dynamics reveal distinct electrical signatures for mechanical and thermal inputs. To our knowledge, this represents the first demonstration of thermally and mechanically evoked electrophysiological activity in an engineered innervated human skin construct. This model enables controlled investigation of sensory encoding and establishes a biologically grounded and experimentally controllable interface between engineered tissues and electrophysiological sensing technologies with potential application in bioinspired tactile sensing, neural interfaces, and sensory restoration.

## Graphical abstract

An innervated human skin equivalent is integrated with high‐density microelectrode arrays to reveal how engineered free nerve ending–like structures translate touch and temperature into electrical codes. Mechanical indentation and thermal stimulation engage the engineered somatosensory circuitry and evoke distinct firing and waveform signatures, establishing a controllable in vitro‐relevant platform for decoding somatosensation and advancing bioinspired sensory interfaces.

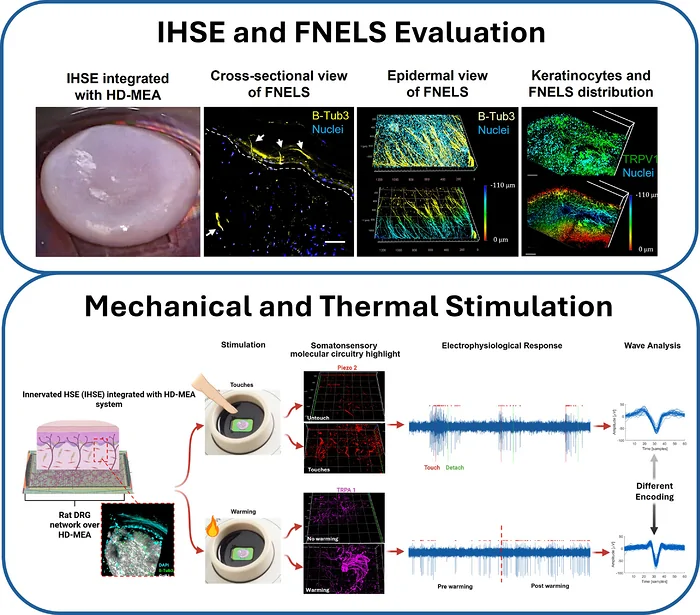

## 1 Introduction

The somatosensory system enables the detection and discrimination of mechanical and thermal stimuli through specialized peripheral sensory neurons that innervate the skin [1, 2, 3]. These neurons transform physical inputs into patterns of electrical activity that are interpreted by the central nervous system, eliciting sensations such as touch, temperature, and pain [4]. Major advances over the past two decades have identified key molecular mediators of sensory transduction, including thermosensitive TRP channels and mechanosensitive Piezo channels [5, 6, 7]. The central role of these molecular sensors was recognized by the 2021 Nobel Prize in Physiology or Medicine awarded to David Julius and Ardem Patapoutian for the discovery of receptors underlying temperature and mechanical sensing in somatosensory neurons [8, 9]. Despite these advances, how peripheral sensory circuits transform physical stimuli into distinct electrical encoding patterns remains incompletely understood [10, 11].

Progress in this area has been constrained by the lack of experimental models that combine native‐like tissue organization with functional electrophysiological readouts [5, 12, 13]. In vivo and ex vivo preparations provide physiological relevance but are limited by accessibility, scalability, and experimental control [14, 15, 16]. Conversely, reductionist in vitro systems, including dissociated neuronal cultures and skin equivalents, typically lack either the structural complexity of native skin or the ability to record electrical activity with sufficient spatial and temporal resolution [12]. As a result, most in vitro studies of human somatosensation rely on chemical stimulation and calcium imaging [12, 17, 18], which report cellular activation but provide limited insight into the electrical dynamics underlying sensory encoding.

Recent advances in tissue engineering have enabled the development of innervated human skin equivalents incorporating keratinocytes, fibroblasts [19], and sensory neurons, in some cases supported by Schwann cells [20, 21]. These models recapitulate key aspects of cutaneous architecture and innervation, including the extension of sensory axons toward the epidermis. However, functional validation has mainly focused on molecular and optical readouts [17, 22], and direct electrophysiological interrogation of sensory responses to mechanical or thermal stimulation at the tissue level remains largely unexplored. To date, mechanically or thermally evoked electrical encoding has been demonstrated primarily in ex vivo human tissues or animal models [14, 23], often with limited reproducibility and experimental flexibility.

Here, we address this unmet need by introducing an innervated, full‐thickness human skin equivalent capable of electrically encoding both mechanical and thermal stimuli. To our knowledge, this represents the first demonstration of thermally and mechanically evoked electrophysiological activity in an engineered Innervated Human Skin Equivalent (IHSE). The model integrates a differentiated epidermis and dermis with sensory neurons and Schwann cells forming free nerve ending‐like structures and is interfaced with high‐density microelectrode arrays to enable non‐invasive, high‐resolution electrophysiological interrogation of sensory activity. Using controlled mechanical indentation and localized thermal stimulation through a custom‐manufactured mechatronic testbed, we demonstrate consistent, stimulus‐dependent responses whose waveform morphology and temporal dynamics reveal distinct encoding signatures for mechanical versus thermal inputs. Beyond providing mechanistic insight into peripheral sensory processing, this model establishes a reproducible in vitro testbed for the investigation of somatosensory encoding and for the development and validation of bioinspired tactile sensors, neural interface technologies, and strategies for sensory restoration and human–machine interaction.

## 2 Result

### 2.1 IHSE Molecular Circuitry is Responsive to Mechanical and Thermal Stimulation

To establish the IHSE platform, a bottom‐up fabrication strategy was followed, as detailed in the Materials and Methods section and summarized in Figure S1. Fibroblasts seeded onto porous gelatin microcarriers formed dermal microtissues that subsequently self‐assembled into Human Dermal Equivalents (HDEs) [24], which were then colonized by sequentially seeded Schwann cells and keratinocytes. Submerged and air‐liquid interface culture conditions promoted the formation of a fully stratified epithelium, giving rise to a Human Skin Equivalent (HSE) characterized by an endogenous dermal compartment enriched with Schwann cells, topped by a differentiated epidermis. The HSE was then integrated with rat DRG sensory neurons cultured on a High‐Density Microelectrode Array (HD‐MEA), yielding the final Innervated Human Skin Equivalent (IHSE). As shown in Figure S1, the resulting platform displayed the expected structural hallmarks of both the dermal and epithelial compartments, together with an organized innervation network and Schwann cell distribution, confirming the successful generation of the IHSE.

To demonstrate the functional activation of the somatosensory circuitry in vitro within the IHSE model, we applied mechanical and thermal stimuli to elicit peripheral sensory response and assessed the corresponding activation of mechano‐ and thermo‐sensitive molecular markers. A schematic representation of the IHSE, highlighting the Free Nerve Endings‐Like Structures (FNELS) is shown in Figure 1 together with the readouts of both the mechanical and thermal stimulations.

**FIGURE 1:**
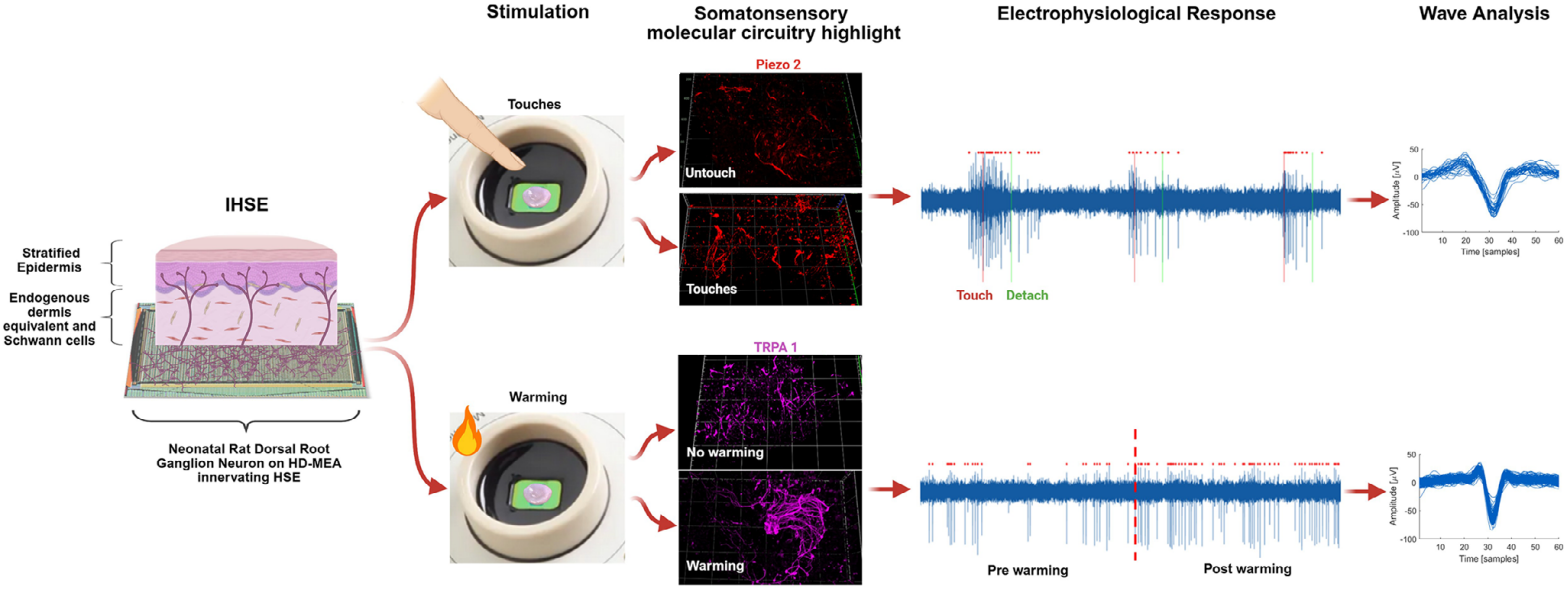
Schematic representation of the experimental workflow. Starting from the Innervated Human Skin Equivalent (IHSE), manufactured according to the protocol previously established [21] (Figure S1), we apply mechanical and thermal stimulation. Immunofluorescence analysis is then used to confirm successful activation of the somatosensory circuitry. Following verification, waveforms are extracted from the electrophysiological traces recorded during stimulation to compare the features of response in the two stimulation conditions.

The effective activation of the somatosensory in vitro circuitry was assessed through immunofluorescence of key ionic channels involved, while the electrophysiological response and wave analysis were further used to characterize the functional response. As reported in literature [25, 26], moderate thermal and mechanical stimuli activate the release of signaling molecules, which, in turn, leads to the overproduction of specific ionic channels in somatosensory neurons. Therefore, we focused our analysis on the FNELS localized within the IHSE, shown in macroscopic view in Figure 2a, with particular attention to the epithelial compartment, examined both in cross‐section (Figure 2b) and through 3D reconstruction (Figure 2c). By using anti‐β‐Tubulin III (Figure 2c,d) and anti‐keratin 10 (Figure 2d) antibodies, we assessed the spatial relationship between FNELS and keratinocytes in the epidermal compartment.

**FIGURE 2:**
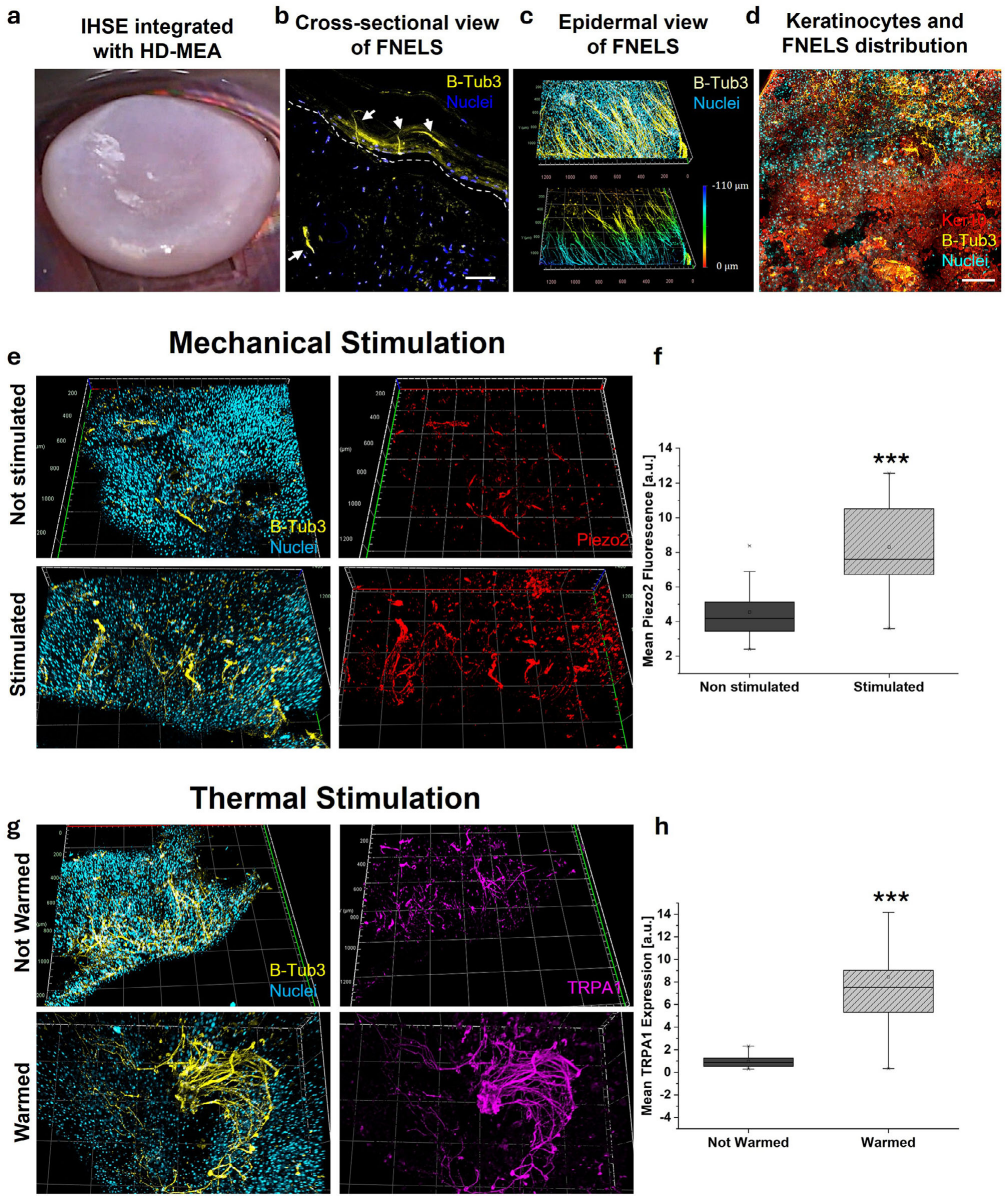
Immunotypization analysis of IHSE responsiveness to mechanical and thermal stimulation. (a) Macroscopic image of the IHSE integrated on the HD‐MEA surface, showing that the HSE covers most of the active area. (b) Immunofluorescence image of a histological section stained with anti‐β‐Tubulin III (yellow) and DAPI (blue), identifying FNELS within the dermis and extending toward the epithelium. (c) 3D‐Reconstruction of a 110‐µm volume beneath the epithelium, with depth‐coded neurites identifiable as originating from the basal region of the IHSE. (d) Tile‐scan of immunofluorescence analysis using anti‐β‐Tubulin III (yellow) and anti‐Keratin 10 (red) with DAPI (light blue), allowing for co‐localization of the keratinocytes and the neurites. (e) Immunofluorescence analysis using anti‐β‐Tubulin III (yellow) and anti‐Piezo2 (red) with DAPI (light blue), comparing non‐stimulated and stimulated samples. (f) Quantification of anti‐Piezo2 fluorescence normalized to anti‐β‐Tubulin III fluorescence, showing higher expression in stimulated compared to non‐stimulated samples. (g) Immunofluorescence analysis using anti‐TRPA1 (magenta) and anti‐β‐Tubulin III (yellow), comparing non‐warmed and warmed samples to qualitatively assess changes in TRPA1 expression. (h) Quantification of anti‐TRPA1fluorescence normalized to anti‐β‐Tubulin III, indicating higher expression following thermal stimulation.

On the other hand, the anchorage of the HSE to the underlying DRG neurons was evaluated through time‐lapse transmitted light microscopy (Figure S2a), while innervation patterns were assessed by 3D reconstruction of the bottom dermal side (Figure S2b) and epidermal side (Figure S2c) of the IHSE. From these reconstructions, the density of innervation was quantified by counting the number of neurites crossing into the epithelium, yielding a mean value of 34.7 ± 15.4 neurites/mm^2^. Additionally, by staining paraffin sections with anti‐keratin 14, anti‐β‐Tubulin III, and using Second Harmonic Generation, we determine the respective position of the basal layer of the epithelium, the fibroblast‐generated stroma, and the innervating neurites (Figure S2d). Direct interactions between neurites and keratinocyte nuclei were also observed (Figure S2e), with the epithelium remaining well‐structured throughout the process (Figure S3a). Finally, several immunofluorescent stains on paraffin sections confirmed the presence of neurites within the dermal compartment (Figure S3b–g). To quantify the response to mechanical stimulation, the mean fluorescence intensity of the anti‐Piezo2 antibody signals between mechanically stimulated and non‐stimulated samples was compared (Figure 2e). We found an increase in the mean fluorescence value from 4.55 ± 1.47 in not stimulated sample to 8.29 ± 2.63 (Figure 2f, *p* < 0.001) in the stimulated ones. A similar procedure was adopted for the analysis of the TRPA1 channel following thermal stimulation (Figure 2g), showing a mean fluorescence increase from 0.95 ± 0.48 to 8.43 ± 5.30 (Figure 2h, *p* < 0.001). The observed overproduction of mechano‐ and thermo‐sensitive ion channels indicates that the IHSE construct is molecularly equipped to detect both mechanical and thermal inputs. This provides a solid mechanistic basis for the electrophysiological characterization presented in the following section and supports the expectation that the recorded signals arise from bona fide activation of the in vitro somatosensory circuitry.

### 2.2 IHSE Electrical Circuitry is Responsive to Mechanical Stimulation

IHSE models exhibited consistent electrophysiological activity, as confirmed by the analysis of spontaneous network signals (Figure S4). As reported in our previous work [21], the IHSE was integrated into the active area of the HD‐MEA surface (Figure S4a), where the majority of detectable spikes were recorded prior to the stimulation experiments (Figure S4b,c). Quantitative assessment of spontaneous electrical activity revealed that morpho‐functional organization (Figure S4d) was consistent with our previous results [21], supporting the presence of a viable and functionally integrated sensory network connected to the previously identified FNELS. All collected electrophysiological parameters are reported in Figure S4e. After establishing the baseline electrophysiological integrity of the IHSE sensory network, mechanical and thermal stimulation were performed.

During the mechanical stimulation (Figure 3a), the electrophysiological activity of the IHSE was monitored, with the response of the system being directly correlated with the indentation. Single‐channel analysis was also performed (Figure 3b) for both physiological validation and waveform extraction (Figure 3c) to be used in waveform comparison across different simulations.

**FIGURE 3:**
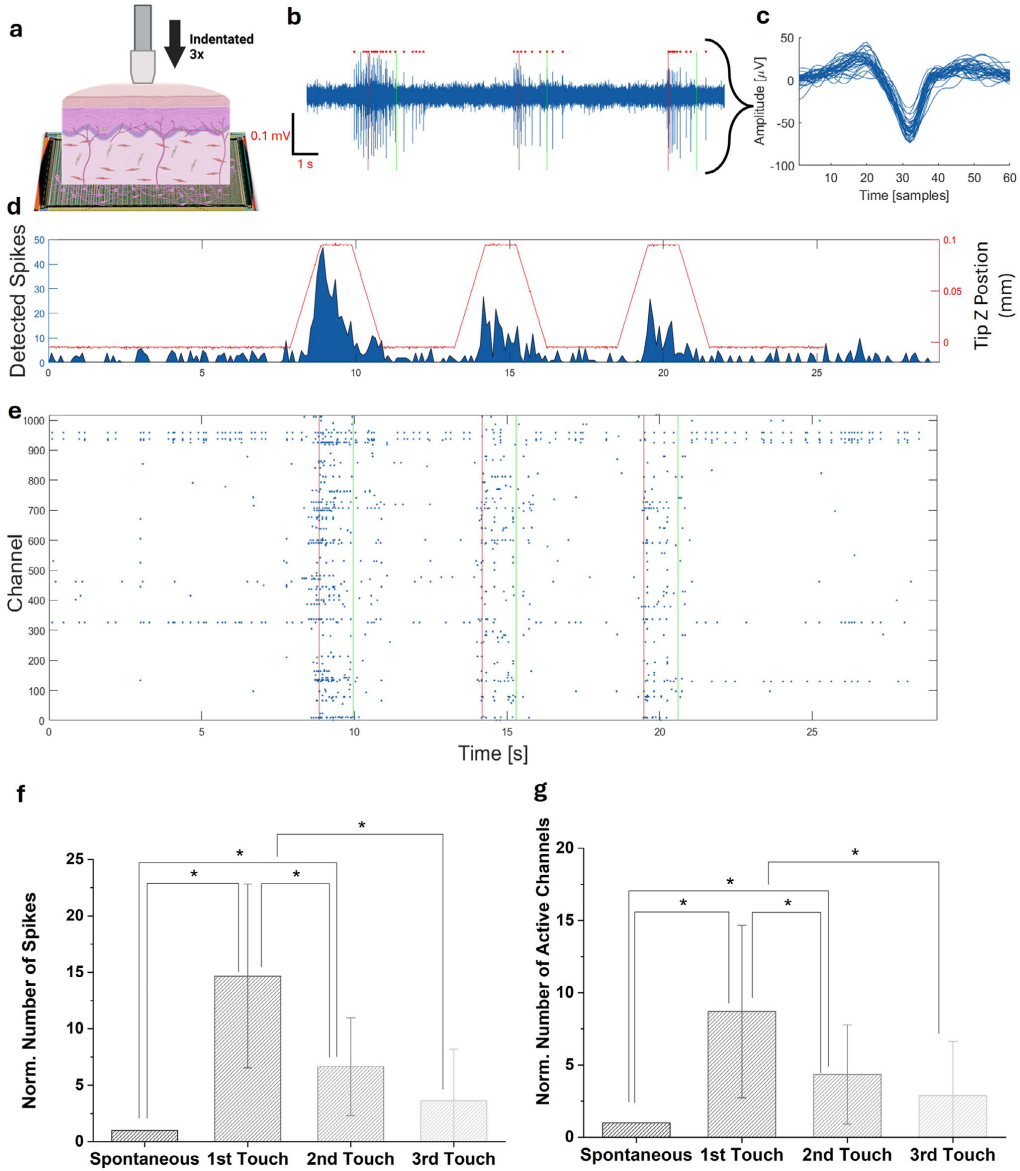
Electrophysiological response of IHSE to mechanical stimulation (a) Schematic representation of the experiment: three 100‐µm indentations (or “touches”) are applied to the surface of the IHSE, with 1 second of stimulation and 1 s intervals between stimulations. (b) Example electrophysiological trace during mechanical stimulation: red line marks indentation onset, while green line indicates tip detachment. (c) Overlaid detected spikes, aligning them by using the spike timestamp as a common reference point in order to obtain an exemplary spike profile for later comparison. (d) Representative plot of detected spikes count (blue) overlaid with the tip position (red), illustrating correlation between mechanical stimulation and firing rate increase. (e) Representative raster plot aligned to tip contact (red) and tip detachment (green), showing an increase in the number of active electrodes. (f) Quantification of mean normalized number of spikes (*n* = 5). Significant differences are observed between spontaneous activity and the first two touches (*p* < 0.05), but not between spontaneous activity and the third touch (n.s.). A significant difference between the first and the third touch indicates a progressive change in responsiveness. (g) Quantitative analysis of the mean normalized number of active channels (*n* = 5). Significant differences are detected between the spontaneous activity and the first two touches (*p* < 0.05), but not between the spontaneous activity and the third touch (n.s.), supporting the previously established conclusions.

By analyzing the overall response of the system in terms of the number of detected spikes (Figure 3d) and of active electrodes (Figure 3e), two behaviors are noticeable: i) a significant increase in both parameters during the indentation of the IHSE; ii) a progressive reduction following consecutive stimulations. Quantification of the parameters was performed by normalizing all values with respect to the spontaneous activity recorded before stimulation. The number of detected spikes is 14.68 ± 8.14 times the spontaneous value during the first touch, 6.64 ± 4.33 during the second, and 3.62 ± 4.57 during the third, while the number of active electrodes is 8.70 ± 5.96 during the first touch, 4.34 ± 3.43 during the second, and 2.88 ± 3.75 during the third. Significant differences were observed in both the number of detected spikes and the active channels (Figure 3g, *p* < 0.05) between spontaneous activity and the first two touches (Figure 3f, *p* < 0.05), while no significant differences were observed between spontaneous and the third touch for both parameters. The progressive decrease in both parameters may be dependent on the adaptation of the IHSE to repetitive stimulation or to progressive desensitization.

A similar protocol was adopted for longer 5 s indentations (Figure S5a). However, prolonged mechanical loading resulted in partial detachment between the anchored neuronal network and the HSE, as observable from both single‐electrode analysis and the marked reduction in detected spikes and active electrodes (Figure S5b,d,e). While this experiment provided useful information, due to the damaging of the in vitro electrical circuitry of the sample, it was not possible to obtain enough results for a robust characterization of the behavior. Nonetheless, the observed behavior is still proportional to the length of the stimulation, as observable in single‐electrode analysis and overall parameters (Figure S5b,d,e).

To demonstrate that the recorded response was not a possible artifact due to the direct force transmission to the electrodes during IHSE indentation, a control experiment was performed by indenting an HSE model without neurons placed on the HD‐MEA surface (Figure S6a). No spikes were detected in response to indentation (Figure S6b), confirming that the recorded activity was not due to mechanical stimulation alone. Moreover, to exclude that the response was caused by the compression of the neuronal somas covered by the IHSE, the same experimental protocol was performed on an IHSE cocultured only for 24 h, which was an insufficient time for the formation of FNELS [21] (Figure S6c). In this case, no change in the number of detected spikes was detected (Figure S6d) compared to the consistent response observed in fully developed IHSE samples (Figure S6e,f), further confirming that the electrophysiological response was a direct consequence of a mature and fully functional developed IHSE model.

### 2.3 IHSE Electrical Circuitry is Responsive to Thermal Stimulation

In contrast to mechanical stimulation, where the analysis interval was well defined, identifying the optimal time window for thermal stimulation analysis required additional modeling to understand the heat diffusion inside the model. To this end, a simulation based on the Finite Element Method (FEM) was adopted to analyze thermal conduction.

Starting from representative video frames of the thermal stimulation (Figure S7a), a simplified geometry was created to identify relevant spatial regions (Figure S7b) and define the corresponding domains for FEM‐based simulations (Figure S7c). Thermal simulations were run with a tip temperature of 45°C for warm and 10°C for cold stimulation, which were in the range of previously simulated in vivo with the mechatronic testbed [27], with other domains set at room temperature (25°C). Under warm stimulation, the model predicted a steady‐state temperature of 42°C in the upper layer of the epithelium, 41°C in the lower layer of the epithelium, and 37.5°C at the bottom of the dermis, where the neuronal bodies were located. Within the first 5 s of stimulation, the temperature reached 38°C in the upper layer of the epithelium (13°C increase compared to initial conditions), 35°C in the lower layer, and 28°C in the deep dermis (+3°C relative to baseline). After 60 s of warming, temperatures stabilized at 40°C, 39°C, and 33.5°C (Figure S7d). These simulations indicate that within the first 5 s following contact between the IHSE and the heated tip, the electrophysiological signals primarily reflect thermal activation of the innervated epithelium rather than temperature increases at the level of neuronal somas. Cold stimulation exhibited an analogous spatiotemporal pattern. Steady‐state temperatures reached 13°C, 14°C, and 17.5°C across the anatomical regions identified. After 5 s of stimulation, temperatures were respectively 16°C, 17.5°C, and 23°C, while after 60 s, the values were 14°C, 14.8°C, and 18.5°C (Figure S7e). Together, these results confirmed that the first 5 s were still the optimal time window for detecting changes in electrical activity.

Guided by the insights gained from the thermal simulations, we then analyzed single‐electrode traces, spike detection rate, and active channel counts during warm stimulation (Figure 4a–e). Quantitative analysis confirmed the following trend: the number of detected spikes after stimulation increased by 61% compared to the number of spikes in spontaneous activity (Figure 4f, *p* < 0.001), and the number of active channels increased by 40% (Figure 4g, *p* < 0.001). Comparison of pre‐stimulation activity with the activity recorded after 60 s of stimulation further revealed increases of 15% and 26%, respectively (Figure 4h, *p* < 0.001). Analogous analyses were performed for the cold stimulation (Figure 5a), including single electrode recordings, to observe the change in activity (Figure 5b), and waveshape inspection, to confirm the physiological nature of the response (Figure 5c). From the overall analysis, the change in the number of detected spikes (Figure 5d) is visible but less pronounced compared to the warm stimulation, with the same pattern noticeable in the active electrodes (Figure 5e). Quantitatively, spike counts increased by 14% in the first 5 s following stimulation (Figure 5f, *p* < 0.01) while the apparent increase in active electrodes did not reach statistical significance. By comparing the number of detected spikes before stimulation and 60 s after stimulation, a slight reduction of 3% and 13% compared to the spontaneous activity was observed (Figure 5h), indicating a time‐dependent decline significantly different from the initial 5 s response window.

**FIGURE 4:**
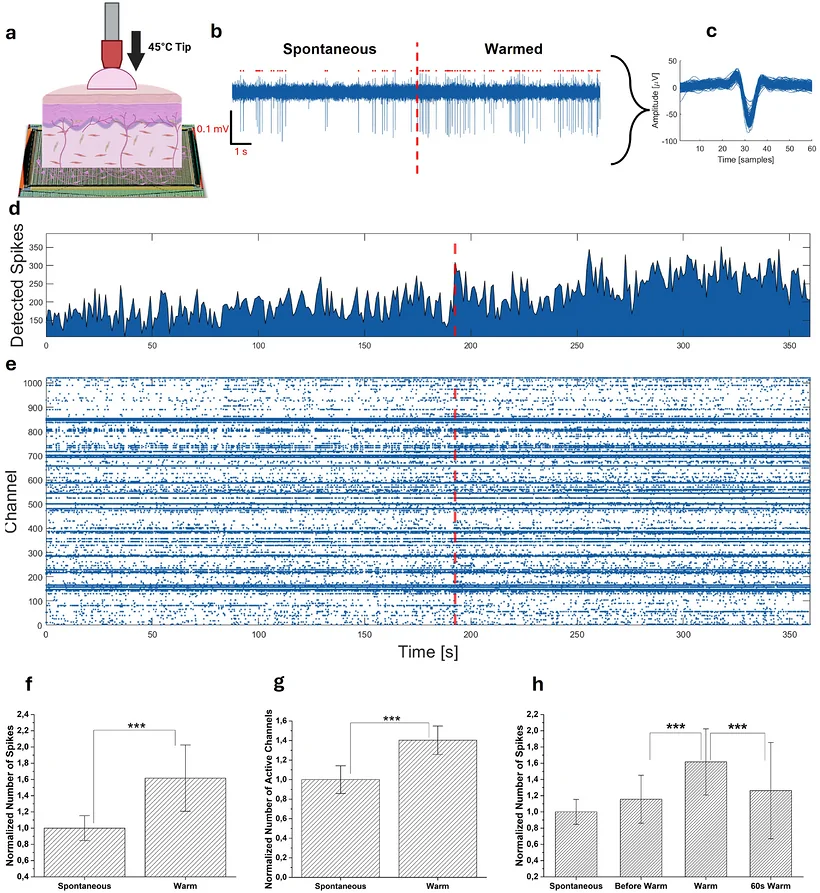
High Temperature‐Evoked Electrical Activity of IHSE (a) Schematic representation of the warm thermal stimulation experiment: the thermal tip at 45°C is put in contact with the IHSE at the 180 s timestamp. (b) Exemplary recorded signal from one of the electrodes during the thermal stimulation experiment: red dotted line indicates the contact time between the tip and the IHSE. (c) Overlapping of all the detected spikes, aligning them using the spike timestamp as a common time point in order to obtain an exemplary spike profile. (d) Exemplary plot of the recorded number of spikes, with the dotted line indicating the contact time between the tip and the IHSE. (e) Exemplary raster plot, with the dotted line indicating the contact time between the tip and the IHSE. (f) Quantitative analysis of the mean normalized number of spikes (*n* = 3), where a significant difference is observed between spontaneous activity and warm stimulation (*p* < 0.001). (g) Quantitative analysis of the mean normalized number of spikes (*n* = 3), where a significant difference is observed between spontaneous activity and warm stimulation (*p* < 0.001). (h) Quantitative analysis of the means of normalized number of spikes comparing the 5 s immediately before contact with the thermal tip and after 60 s of thermal stimulation. Both are significantly different compared to the first 5 s following thermal stimulation.

**FIGURE 5:**
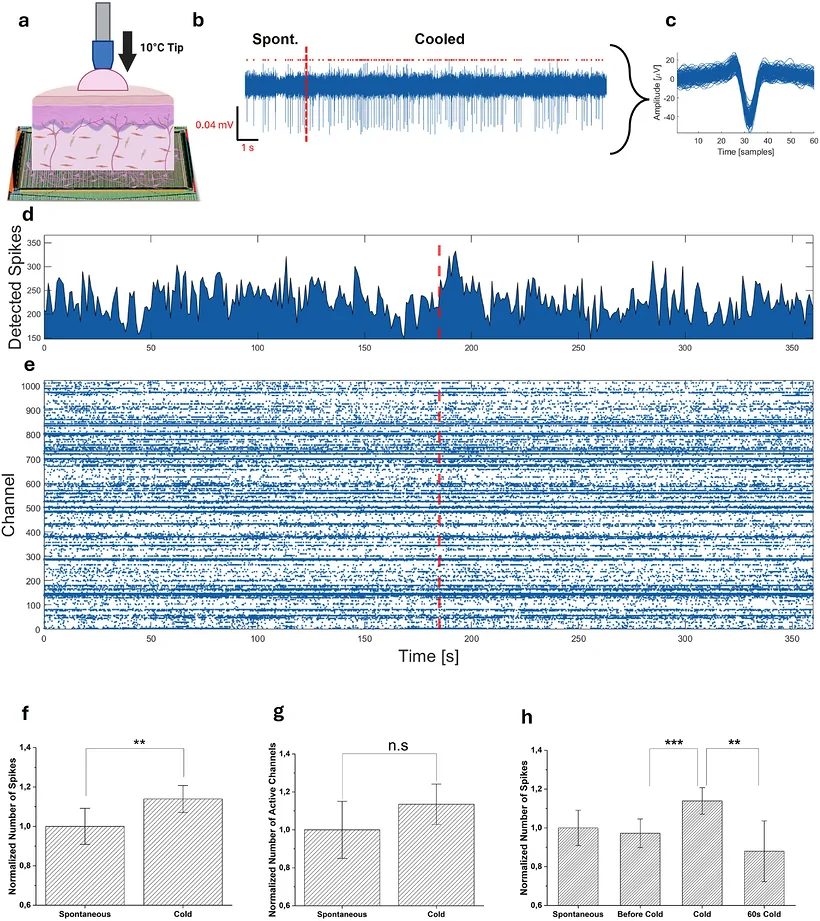
Cold Temperature‐Evoked Electrical Activity of IHSE. (a) Schematic representation of the cold thermal stimulation experiment: the thermal tip at 10°C is put in contact with the IHSE at the 180 s timestamp. (b) Exemplary recorded signal from one of the electrodes during the cold stimulation experiment: red dotted line indicates the moment of contact between the tip and the IHSE. (c) Overlapping of all the detected spikes, aligning them through the spike timestamp to obtain an exemplary spike profile. These will be used for the waveform comparison. (d) Exemplary plot of the recorded number of spikes, with the dotted line indicating the contact time between the tip and the IHSE. (e) Exemplary raster plot, with the dotted line indicating the contact time between the tip and the IHSE. (f) Quantitative analysis of the mean normalized number of spikes (*n* = 3), where a significant difference is observed between spontaneous activity and cold stimulation (*p* < 0.01). (g) Quantitative analysis of the mean normalized number of spikes (*n* = 3), where no significant difference was observed. (h) Quantitative analysis of the mean normalized number of spikes comparing the 5 s immediately before contact with the thermal tip and after 60 s of thermal stimulation. Both are significantly different compared to the first 5 s following thermal stimulation.

As for a mechanical test, to exclude artifacts arising from tip contact during thermal stimulation, a control experiment was conducted by setting the tip at 28°C, close to room temperature (Figure S8a). No significant variations in single electrode activity were observed (Figure S8b,c). On the other hand, a slight qualitative increase in the number of spikes detected and active channels was observed (Figure S8d,e); however, quantitative analysis across all the samples indicates no significant changes (Figure S8f,g).

### 2.4 Electrical Waveform Train Analysis Reveals Divergent Mechanical and Thermal Patterns

After evaluating the overall parameters for mechanical, warm, and cold stimulations, all the extracted waveforms were compared to determine the degree of correlation with each other, with a representative comparison shown in Figure 6a. The highest Pearson's coefficient, calculated as the mean of the correlations of all the possible couples of waveforms of two stimulations, is found between warm and cold stimulation, with a value of 0.88 ± 0.09. The comparison between mechanical versus warm and mechanical versus cold stimulations yielded coefficients of 0.84 ± 0.11 and 0.82 ± 0.13, respectively. While these values indicate a strong correlation, some slight waveform differences can be observed. The morphological waveform parameters indicated in Figure 6b were calculated for each type of stimulation, with the mean values shown in the radar plot in Figure 6c and the quantitative analysis and statistical values reported in Tables 1 and 2. As visible from the shapes of the polygons in the radar plot, while warm and cold produced comparable waveform signatures, mechanical stimulation displayed significant differences in several parameters. Specifically, five features showed the most pronounced differences: interspike interval, half‐peak width, trough, amplitude, and depolarization and repolarization slopes. Once the most important parameters for the encoding of the information related to the stimulation were determined, Principal Components Analysis (PCA) was performed to determine the presence of clustering, which would help in differentiating the stimulations from each other. The results shown in Figure 6d indicate partial clustering of waveforms according to stimulation type, showing separation between mechanical and thermal responses, while it was not possible to discriminate between warm and cold stimulations due to their high degree of waveform similarity. Mahalanobis distances between the centroids of the three clusters were calculated to quantify cluster separation and used for statistical comparisons. Although inter‐cluster distances were small, the distributions differed significantly. In particular, Mechanical–Cold and Mechanical–Warm differed significantly (*P* < 0.001), whereas the Cold–Warm comparison was not significant.

**FIGURE 6:**
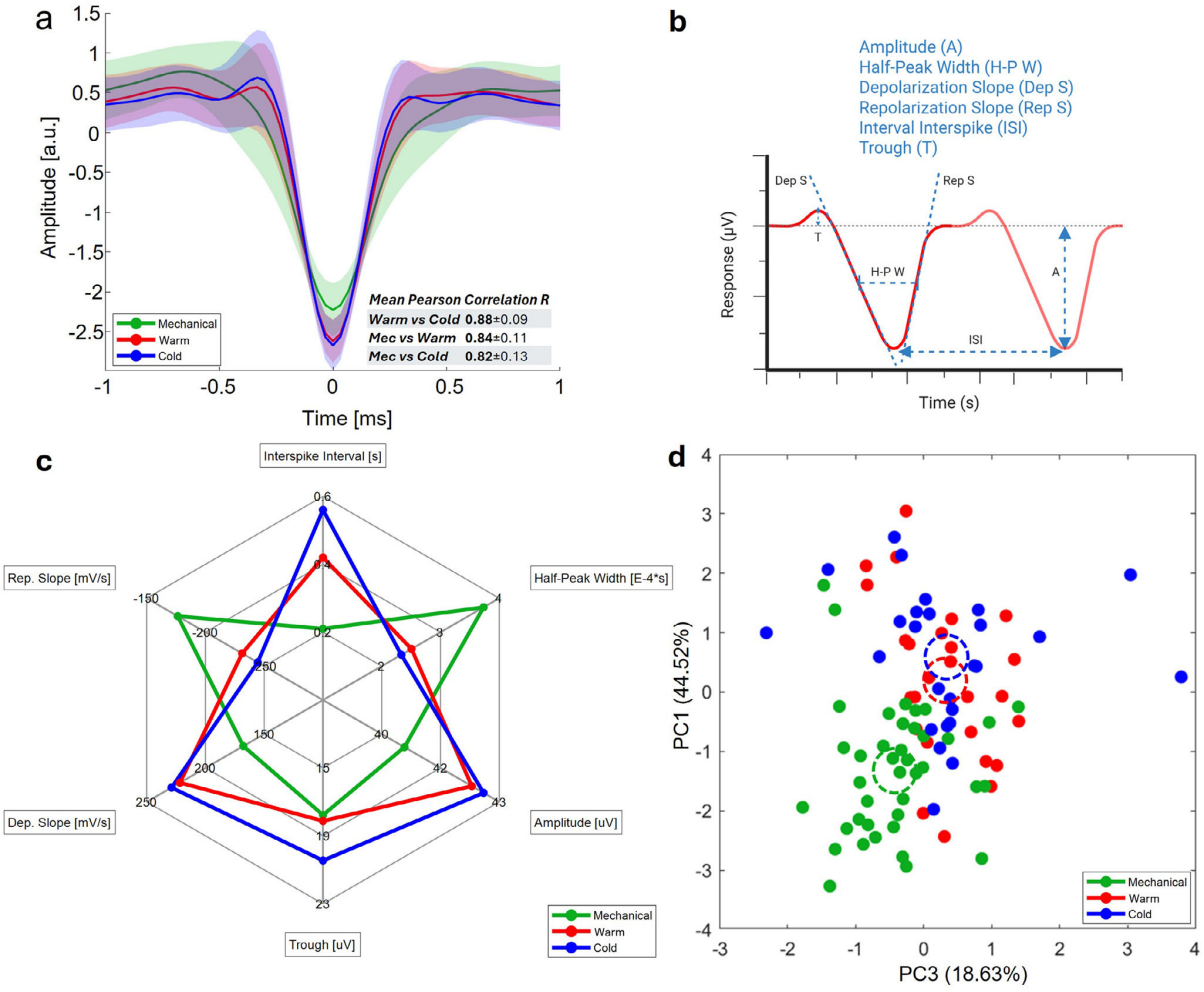
Comparison of Electrical Stimulation Waveforms. (a) Comparison among mean waves of each stimulation (green‐mechanical; red‐warm; blue‐cold) with the reported values for Pearson's Correlation between each couple of stimulations. Shaded areas correspond to the standard deviation of each average waveform. (b) Schematic representation of the morphological features of the train of waves used for the subsequent analyses. (c) Radar plot representing the morphological properties. Qualitatively, the mechanical and thermal stimulation are different from each other, while cold and warm are quite similar. (d) Graph of the PCA performed using the morphological parameters with the highest significant differences. Mechanical stimulation results form a specific cluster compared to the thermal stimulation. Mahalanobis distance between the centroids clusters was evaluated to determine whether a statistical difference was present. (Mechanical – Warm: *p* < 0.001, Mechanical – Cold: *p* < 0.001, Warm‐Cold: N.S.). Dotted color‐coded lines indicates the location of each cluster centroid.

**TABLE 1:** Wave Parameters calculated as Mean + STD for each morphological feature.

	Interspike interval (s)	Half‐peak width (10^4^*s)	Amplitude (uV)	Trough (uV)	Depolarization slope (mV/s)	Repolarization slope (mV/s)
Mechanical	0.16 ± 0.14	3.80 ± 1.00	40.58 ± 12.30	20.45 ± 6.17	167.79 ± 105.24	−176.39 ± 100.55
Warm	0.44 ± 0.28	2.88 ± 0.62	42.30 ± 9.96	17.77 ± 4.52	221.69 ± 103.90	−231.25 ± 129.28
Cold	0.51 ± 0.23	2.76 ± 0.54	42.63 ± 10.44	18.12 ± 3.48	228.89 ± 112.76	−244.46 ± 110.50

**TABLE 2:** Statistical analyses of each wave parameters, compared pairwise and with the respective *P* Values. Significant differences are observable between mechanical and thermal stimulations.

	Interspike interval	Half‐peak width	Amplitude	Trough	Depolarization slope	Repolarization slope
**Mechanical vs Warm**	<0.05	<0.001	N.S.	<0.001	<0.001	<0.001
**Mechanical vs Cold**	<0.01	<0.001	N.S.	<0.001	<0.001	<0.001
**Warm vs Cold**	N.S.	<0.05	N.S.	N.S.	N.S.	N.S.

## 3 Discussion

Results demonstrate that the IHSE electrically encodes both mechanical and thermal stimulation through consistent, stimulus‐dependent patterns of electrical activity (Figures 3, 4, 5, 6), enabling direct interrogation of peripheral sensory encoding at the tissue level within a controlled in vitro construct.

Building on the structural and molecular validation of the IHSE, including the formation of free nerve ending‐like structures within the epidermal compartment (Figures 1 and 2a–d, Figures S1, S2, and S4), we next interpret how this architecture supports functional sensory encoding compared with existing in vitro skin models. Functional validation of existing innervated skin models has largely relied on chemical stimulation and optical readouts such as calcium imaging [12, 14, 17, 28]. These approaches provide valuable insight into cellular activation, albeit they do not capture the temporal structure of neuronal firing that underlies sensory encoding. With few exceptions— most notably an ex vivo mechanically responsive human hair follicle preparation [15]—direct electrophysiological interrogation of mechanically or thermally evoked sensory responses in human skin constructs has remained largely unexplored. The limited reproducibility and structural complexity of ex vivo preparations further emphasize the need for scalable in vitro alternatives. To our knowledge, this represents the first demonstration of mechanically and thermally evoked electrophysiological activity in an engineered innervated human skin construct. This capability provides direct access to sensory transduction dynamics in a controlled in vitro environment, which has not been achievable with previous skin models.

Mechanical stimulation of the IHSE elicited consistent increases in firing rate and spatial recruitment of active electrodes that scaled with indentation and adapted over repeated stimulation (Figure 3d–g), consistent with analogous measurements in peripheral sensory neurons in vivo [29]. These findings indicate that the model reproduces key functional properties of peripheral mechanosensory circuits. The absence of mechanically evoked activity in non‐innervated or immature co‐cultures lacking FNELS (Figure S6) further confirms that these responses arise from tissue‐level sensory transduction rather than direct neuronal compression or electrode artifacts.

The progressive decrease in electrophysiological response over successive stimulations may be dependent on the adaptation of the IHSE to repetitive stimulation or to desensitization of the IHSE to stimulation over time, which would be consistent with nociceptive desensitization upon repeated stimulation—a physiologically documented phenomenon across multiple nociceptor subtypes [30]—rather than neuronal degeneration. An analogous pattern of declining firing rate accompanied by receptor upregulation was previously observed in the same model following TRPV1‐mediated activation by capsaicin and was interpreted as a compensatory response to stimulation‐induced desensitization [21]. The morphological integrity of axons visible in Figure 2e, which shows no fragmentation, further argues against neuronal injury as the underlying cause.

Guided by FEM‐based thermal conduction simulations that defined the spatiotemporal features of temperature within the IHSE (Figure S7), we interpret the early electrophysiological responses to thermal stimulation as arising from epithelial‐level transduction rather than direct heating of neuronal soma. Consistent with this interpretation, warm stimulation induced a marked increase in spike rate and active channels (Figure 4f–h), whereas cold stimulation produced smaller but detectable responses (Figure 5f–h), and room‐temperature controls showed no significant changes (Figure S8). Comparison between the three thermal ranges (10°C, 28°C and 45°C) is presented in Figure S9. The statistical analysis confirms that the system response is significantly different to 10°C and 45°C stimulations, while the cold and room temperature are close enough in values to not be significantly different. This may indicate that our setup, which works at room temperature, may be less capable of measuring and detecting cold stimulations compared to warm stimulations. This is something that may be addressed in future work on the topic.

While firing rate and spatial recruitment establish stimulus responsiveness (Figures 3, 4, 5, Figure S10), the waveform and temporal analyses in Figure 6 reveal how the IHSE encodes stimulus modality at the electrical level. Mechanical stimulation produced waveform morphologies and interspike interval distributions that were distinct from those elicited by thermal stimuli (Figure 6a–c), enabling partial separation of modalities by principal component analysis (Figure 6d). The importance of interspike interval metrics in separating stimulus classes aligns with established models of temporal coding in mechanoreception and thermoception [31, 32], yet such discrimination has not previously been demonstrated, to our knowledge, within an engineered in vitro human tissue system.

The encoding features identified electrophysiologically are supported by stimulus‐dependent modulation of canonical sensory transducers. Semiquantitative immunofluorescence analysis revealed stimulus‐dependent overproduction of Piezo2 upon mechanical stimulation (Figure 2e,f) and TRPA1 upon thermal stimulation (Figure 2g,h), consistent with activation of recognized mechanotransduction and thermotransduction circuitry [25, 26, 33]. Consistent with thermal simulation results, early electrophysiological responses arise predominantly from temperature changes at the epithelial–neuronal interface rather than direct heating of neuronal soma (Figure 4h). Control experiments excluding neuronal compression or electrode artifacts reinforce the conclusion that recorded signals originate from FNELS‐mediated sensory activation (Figure S6).

The mechanistic interpretation of these results is supported by four independent and convergent lines of evidence: (i) stimulus‐specific molecular upregulation of the canonical transducers Piezo2 and TRPA1, demonstrating selective engagement of the mechanotransduction and thermotransduction pathways respectively; (ii) signal dependence on mature epidermal innervation, confirmed by two structural negative controls showing that neither a non‐innervated HSE nor an immature 24‐h co‐culture generate evoked responses; (iii) stimulus‐modality specificity, confirmed by the absence of significant electrophysiological changes upon room‐temperature probe contact; and (iv) FEM‐confirmed spatial localization of the thermal transduction event to the epidermal level, where TRP channels reside, predicting that heat would require minutes to reach neuronal somas in the absence of FNELS‐mediated transduction. Furthermore, the TRP‐expressing sensory circuitry of the IHSE has been independently validated at the pharmacological level in prior work from our group: topical capsaicin, a selective TRPV1 agonist with no other known targets in this circuit, elicited consistent FNELS‐mediated electrophysiological responses in two independent publications using the same model system and cell sources [17, 21]. The present study extends this pharmacologically validated circuitry to physically evoked stimuli and provides convergent evidence that the recorded signals reflect bona fide activation of the in vitro somatosensory apparatus through the expected molecular pathways. A survey of the literature indicates that, to the best of our knowledge, a direct and detailed comparison of in vivo electrophysiological responses to thermal versus mechanical stimulation has not been systematically performed. Although qualitative differences in response properties have been reported [34], a quantitative waveform‐based comparison remains missing. Nonetheless, differences in spike shape and firing patterns are expected, given the activation of distinct membrane channels—such as TRP and Piezo channels [5, 6]—which are selectively responsive to specific stimulus modalities.

Moreover, several in vivo studies have classified cutaneous afferents based on conduction velocity, waveform morphology, and stimulus selectivity, particularly in small fibers innervating human skin [3, 35, 36, 37, 38]. Therefore, the differences we observe in morphological spike parameters and firing rates across stimulation modalities are consistent with established in vivo findings. A more direct validation, however, is not currently feasible due to the absence of comparable in vivo studies performing equivalent cross‐modal waveform analyses.

After establishing that our model, similarly to in vivo systems, can discriminate between mechanical and thermal stimuli, we compared our dataset with publicly available recordings of rat sciatic nerve activity during mechanical stimulation of the paw using a 100 g von Frey Filament [39]. The comparison yielded a moderate Pearson correlation coefficient of 0.58 ± 0.17 (Figure S11).

To confirm that this degree of moderate correlation is dependent on the morphology of the waveforms and not on other spectral properties, surrogate waveforms were generated from the starting set according to the literature [40], having the same spectral density of the original signal, but with randomized amplitude, destroying the shape of the signal. The mean Pearson correlation coefficient between the surrogate waveforms and the in vivo waveforms was evaluated to be 0.02 ± 0.37 (Figure S11b). This also allowed us to determine if the two distributions of coefficients were significantly different, which was confirmed with *p* < 0.001 (Figure S11c). While the observed differences between in vivo and in vitro waveforms likely reflect both experimental factors (e.g., stimulation paradigms and recording configurations) and biological factors (e.g., the greater structural and cellular complexity of in vivo systems), comparison with the surrogate indicates that the correlation captures signal morphology features intrinsically linked to the biological identity of the systems analyzed. Comparison with publicly available in vivo mechanoreceptor recordings revealed moderate similarity (Figure S11a), as expected given differences in species, tissue complexity, and recording modalities. Nevertheless, this comparison demonstrates that the electrical signatures generated by the IHSE fall within a physiologically meaningful range, supporting the usefulness of the model as a bridge between reductionist neuronal cultures and native sensory tissues. Importantly, although a direct one‐to‐one correspondence between the recorded in vitro signals and in vivo peripheral nerve activity cannot yet be established, multiple observations support the electrical signatures' biological plausibility. The signals arise only in the presence of mature free nerve ending‐like structures, disappear in control conditions lacking these structures, and are modulated by stimulus modality, indicating that they reflect functional sensory transduction within the engineered tissue. Although the IHSE might not fully reproduce the complexity of native human skin, the recorded signals arise from biologically grounded transduction mechanisms mediated by TRP and Piezo channels within functional free nerve ending‐like structures. As such, the system captures essential features of peripheral sensory transduction while providing a simplified and experimentally valid testing platform. The interpretation of these Results should be considered in light of several limitations inherent to the current implementation of the IHSE. First, although the IHSE recapitulates key features of free nerve ending‐mediated sensation, it lacks specialized mechanoreceptive end organs such as Meissner, Pacinian, Ruffini corpuscles, and specialized cells, such as Merkel cells, which contribute to fine touch and vibration sensing in vivo [3, 41]. Second, the use of rat dorsal root ganglion neurons innervating human skin tissue introduces interspecies differences that may influence channel expression and firing dynamics. Third, stimulation thresholds were selected based on in vivo data due to the absence of established in vitro benchmarks for human skin equivalents, and further work will be required to refine modality‐specific stimulus ranges. Finally, while waveform‐based analyses reveal clear modality separation, additional computational and experimental studies will be needed to link these electrical signatures to perceptual correlates. In the future, by testing additional temperature and mechanical stimuli to fully map the model sensitivity across different conditions, we could aim to develop analysis systems able to discriminate and identify stimulation from the firing rate patterns and wave parameters, even in the presence of unknown and/or combined stimulations

The current study does not include pharmacological inhibition of ion channels (e.g., antagonists of TRPA1, TRPV1, or Piezo2) as a direct confirmation of receptor‐mediated signal transduction. This reflects the absence of established antagonist protocols validated for three‐dimensional innervated skin models in the literature [20, 42, 43, 44], the documented off‐target limitations of first‐generation antagonists including capsazepine [45, 46] and HC‐030031 [47, 48], and the complete absence of a selective Piezo2 blocker. Development of validated pharmacological inhibition protocols adapted for 3D organotypic constructs, employing highly selective compounds such as A‐967079 for TRPA1 [49] and SB‐366791 for TRPV1 [50], represents a prioritized direction for future work.

Together, the mechanical, thermal, and encoding analyses presented in Figures 3, 4, 5, 6 establish the IHSE as an experimentally valid and controllable platform for probing sensory signal transduction and encoding at the tissue level. From an engineering perspective, the IHSE represents a modular and scalable system for studying sensory encoding at the tissue level. The combination of controlled mechanical and thermal stimulation with high‐density electrophysiological readouts enables systematic exploration of how biophysical parameters are translated into neural signals. This capability is directly relevant to the development of bioengineered sensory interfaces, including tactile and thermal feedback systems for prosthetic devices, neuromorphic skin technologies, and human–machine interfaces. Moreover, the reproducible and non‐invasive nature of the platform makes it suitable for medium‐throughput screening applications targeting nociception and somatosensory modulation, potentially reducing reliance on animal models. Rather than replicating the full physiological complexity of human skin, the IHSE establishes a minimal yet biologically grounded platform in which the transformation of physical stimuli into neural signals can be directly measured and manipulated, bridging biological somatosensation and engineered sensory systems.

In summary, this work establishes an in vitro human skin model that not only detects but electrically encodes mechanical and thermal stimuli through distinct, quantifiable signatures. By linking tissue‐level sensory transduction to neural encoding within a controlled experimental framework (Figures 3, 4, 5, 6 and Figures S5–S11), the IHSE provides a foundation for mechanistic studies of peripheral sensation and opens new avenues for the integration of biological and engineered sensory systems.

## 4 Methods

Human dermal fibroblasts (HDFs) and human dermal keratinocytes (HKE) were extracted from breast skin biopsy as previously reported [21] after the approval of the ethical committee of the University of Naples Federico II and AORN A. Cardarelli (Prot.N. 00019018). Three donors, aged 18–50, were included to account for interindividual variation.

Study design. This study employed an in vitro IHSE to assess electrophysiological responses to controlled mechanical and thermal stimulation.

### 4.1 IHSE Manufacturing

IHSE has been manufactured according to the protocol established in a previous paper from our research group [21]. Briefly, here we report a protocol summary.

#### 4.1.1 Skin Equivalent (HSE) Manufacturing

HDFs were suspended into T‐150 flasks and cultured in Minimum Essential Medium (M2279‐500ML, Merck) supplemented with 20% Fetal Bovine Serum (F7524‐500ML, Merck), 2% Non‐Essential Aminoacids (ECB3054D, Euroclone), 1% Penicillin/Streptomycin (A001‐100ML, HiMedia), and 1% L‐Glutamine (G7513‐100ML, Merck) and used at passages 4–12.

HDE was fabricated according to an established protocol [24]. Human dermal fibroblasts (HDFs) and porous gelatin microcarriers were suspended in a spinner flask to enable progressive HDF seeding within the microcarrier porosities. The HDF‐seeded microscaffolds were cultured under suspension conditions for 9 days, leading to the formation of microtissues. These microtissues were then transferred into maturation chambers (1 mm thickness, 8 mm diameter) and maintained within spinner flasks operating at 60 rpm for up to 6 weeks, resulting in the formation of HDE tissue free of scaffolding material. The culture medium was fully exchanged twice per week during the first half of the procedure and once per week thereafter. Ascorbic acid (50 µg/mL) was added twice weekly throughout the culture period.

The porous gelatine microcarriers were produced through an oil‐water‐oil technique, then sterilized in 100% Ethanol for 24 h, washed thrice with Phosphate Buffered Salines (PBS), and pre‐conditioned with culture medium.

#### 4.1.2 Human Schwann Cells (HSCs) Integration

Human Schwann Cells (HSC, ScienCell, Cat #1700) were seeded on one side of the mature HDEs at a cell density of 200.000 cell/cm^2^. The tissues were cultured in submerged conditions by using 50% Supplemented Schwann Cell growth medium (ScienCell, Cat# 1701) and 50% HDF Growth Medium for 4 days, allowing the HSC to proliferate.

#### 4.1.3 Epidermal Assembly

Human Keratinocytes (HKE) were extracted from a human breast biopsy. HKE were seeded on the same side where HSC were pre‐seeded, using 8 µL of a solution of human fibronectin at 50 µg/mL to enhance the initial adhesion of the epidermal cells. The HDEs were left for 45 min uncovered inside a laminar flow hood surrounded by fibronectin solution to avoid the drying of the HDE. Then, keratinocytes were seeded at a cell density of 200 000 cells/cm^2^ into an 8 µL drop of CnT‐Prime Full Thickness 3D Airlift Medium (FTAL, CellnTec Cat CnT‐PR‐FTAL5) and left for 2 h to adhere. Then, the obtained Human Skin Equivalents (HSEs) were first cultured in submerged conditions for 4 days and then for 12 days at the air–liquid interface inside transwell inserts (Corning, CLS3801‐24EA). The final result has a 100 µm epithelium, with an overall thickness of around 1 mm.

#### 4.1.4 Sensory Neurons Innervation

Dorsal Root Ganglion (DRG) neurons were used to innervate the HSE. Neonatal rat DRG neurons were used due to their robustness, reproducibility, and established compatibility with high‐density microelectrode array recordings. MaxOne Small and Large chips (MaxWell Biosystems) were sterilized and conditioned with R‐DRG culture media according to the manufacturer's instructions. The area of the electrodes was coated with 15 µL of PDL at 0.1 mg/mL concentration for 2 h, washed thrice with sterile deionized water, left to dry under laminar flow until completely dry, and then coated with 8 µL of 0.01 mg/mL laminin for 2 h and 30 min. Coating was aspirated just before seeding neonatal Rat Dorsal Root Ganglion (R‐DRG‐505, Lonza) at a density of 30.000 cells/chip into an 8 µL drop of culture medium. The chips were incubated at 95% humidity, 5% CO_2_, 37°C for 1 h. Cells were seeded and cultured on‐chip using the R‐DRG medium prepared according to the literature [51]. Cells were monitored for 13 days, and electrophysiological recordings were performed after the first week of culture. After 13 days, samples for the 2D culture were maintained as previously described, while samples for 3D DRG+HSE were treated in the following way: medium was delicately removed, HSE was gently placed on the active area of the HD‐MEA, and a small amount of medium was provided to avoid the sample floating. Final medium volumes were 200 µL for small chips and 550 µL for larger chips. Samples were co‐cultured for 9 days with daily, full media exchanges, and uridine (Sigma Cat. No. U‐3003) and 5‐fluoro‐2’‐deoxyuridine were removed (Sigma cat # F0503).

### 4.2 IHSE Stimulation Through Custom‐Made Mechatronic Testbed

#### 4.2.1 Custom‐Made Mechatronic Testbed for IHSE Controlled Stimulation

The testbed used for the mechanical and thermal stimulation, as introduced by Sperduti et al. [27], is mainly composed of: i) a positioning system, ii) a mechanical stimulator, iii) a thermal stimulator, iv) a user interface.

The positioning system consists of a 3‐axis Cartesian arrangement of three linear stages (VT‐80, Physik Instruments) actuated by DC motors and featuring a resolution of 0.5 µm. The linear stages are driven by a positioning controller, namely the multi‐axis driver C‐884.4DC. A 3D‐printed holder was used to simultaneously connect the thermal and mechanical stimulators to the end‐effector of the positioning system (vertical axis).

The mechanical stimulator is based on the 300 C Dual Mode device by Aurora Scientific (ON‐Canada), composed of a motor head whose indenter ends with an interchangeable tip (for our purposes, a cylindrical tip with a diameter of 0.6 mm was used). The indenter can exert a maximum force of 5 N with a resolution of 1 mN. The actual position and the delivered force are both continuously recorded during the experiments to monitor the contact with the IHSE. The mechanical stimulator is driven by a mechanical stimulator controller, which receives desired position commands.

The thermal stimulator is made of a Peltier cell (Adaptive, United Kingdom), connected on one side to a heatsink and on the other side to a custom‐made cylindrical aluminum tip (diameter of 3 mm). The temperature of the tip is regulated by using a ThermoElectric Cooling (TEC) controller by Meerstetter Engineering (TEC‐1091) (Rubigen, Switzerland) based on two NTC (Negative Temperature Coefficient) thermal sensors. The feasible temperature range is 5°C–60°C. Preliminary tests proved stability in temperature control and recording despite contact with IHSE.

The user interface allows the experimenter to control the position and record the force for the mechanical stimulator, to control and monitor the temperature for the thermal stimulator, and to control the position for the positioning system.

In addition to these tools, a camera is added to visually monitor the IHSE on a dedicated display.

A representation of the used setup is provided in Figure S12.

#### 4.2.2 Electrophysiological Recording Protocol

On the day of the experiment, an Activity Scan Assay (MaxLive Software, MaxWell Biosystems) with the checkered configuration was run in order to determine the location with the highest activity. Then, a Network Assay using the “Neuronal Units” option was used to route the 1020 electrodes to the highest activity locations. Default device parameters were used (sampling frequency of 20000 Hz and 300–3000 Hz bandpass filter).

For the thermal stimulation, a 3 min Network Assay was run before the experiment to obtain a baseline value for the firing rate. Then, another 3 min of recording started. Contact between the wet thermal tip (by using culture media) of the mechatronic testbed and the IHSE surface was determined using the DinoLite digital microscope (AM7025X, Dino‐Lite Europe) and recorded for the following data analysis.

For mechanical stimulation, a similar protocol was followed. Before the experiment, tests to determine the relative position of the testbed to the IHSE were conducted by evaluating changes in the force recorded by the instrument. Then, the contact was automatically determined, and an electrical signal was sent to the MaxOne Recording Unit to save the time of contact in the file. Three indentations of 100 µm were performed on the sample, with 1 s for the lowering of the tip, 1 s of contact, and 1 s for the release of the contact. After release, 1 s passed between the beginning of consecutive stimulations.

### 4.3 Immunofluorescence Analysis of IHSE Response to Stimulation and 3D‐Reconstruction

IHSE integrated on HD‐MEA were fixed in 4% paraformaldehyde for 20 min and rinsed thrice in PBS, 24 h after the stimulation. Samples for 3D immunofluorescence were first treated with Triton X‐100 at 0.1% in PBS for 5 min, washed thrice in PBS, and then treated with blocking solution (1% BSA in PBS) for 1 h. After removal of the blocking solution, samples were incubated with the primary antibodies Anti‐β‐tubulin III (Thermo Fisher Scientific, MA1‐118, RRID: AB_2536829, 1:1000), Anti‐Piezo2 (Alomone Labs, APC‐090, RRID: AB_2876842, 1:200), Anti‐TRPA1 (Alomone Labs, ACC‐037, RRID: AB_2040232, 1:500)Anti‐NaV 1.7 (Abcam, ab65167, AB_2301407, 1:1000), diluted in blocking solution, at room temperature for 24 h under orbital agitation (10 rpm). The following day, samples were washed thrice with PBS and incubated for 1 h with the secondary antibodies, diluted 1:500 in PBS: Goat anti‐Mouse IgG (H+L) Alexa Fluor 488 (Thermo Fisher Scientific, A‐11001, RRID: AB_2534069) and Goat anti‐Rabbit IgG (H+L) Alexa Fluor 546 (Thermo Fisher Scientific, A‐11010, RRID: AB_2534077). Samples were washed thrice in PBS, counterstained with DAPI (Thermo Fisher Scientific, 62248, 1:10 000) for 1 h, and washed thrice with PBS. 3D Z‐Stack reconstructions were acquired using an LSM900 confocal microscope (Zeiss).

Second Harmonic Generation (SHG) imaging to detect collagen bundles and tile‐scan imaging were performed using a confocal Leica TCS SP5 II combined with a multiphoton microscope, where the NIR femtosecond laser beam was derived from a tunable compact mode‐locked titanium: sapphire laser (Chameleon Ultra, Coherent). SHG was induced at a wavelength of 840 nm, with emission collected in a window between 415 and 425 nm (for collagen detection).

For quantitative analyses, IMARIS 10.1.1 (Oxford Instruments, RRID: SCR_007370) was used to measure the fluorescence values of Piezo2 or TRPA1 in the area, using β‐tubulin III expression as reference. For density of innervation, neurites were quantified using a reference plane under the epithelium, extracting a volume from the Z‐Stack image, and quantifying the number of neurites crossing the epithelial plane. Density was then evaluated as the number of neurites/area of the plane, similarly to the literature [52].

### 4.4 Histology and Immunofluorescence on Tissue Sections

Samples were fixed in 10% neutral buffered formalin overnight, dehydrated through an ascending ethanol series (75%, 85%, 95% and two changes of absolute ethanol; 30 min each at RT), cleared in xylene, and embedded in paraffin. Longitudinal sections of 7 µm were cut with a microtome (Thermo Scientific HM 355S). For histological analysis, sections were deparaffinized, rehydrated, and stained with Hematoxylin and Eosin (H&E; Bio‐Optica); slides were mounted with Histomount Mounting Solution (Invitrogen) and imaged under bright‐field microscopy (Olympus BX53).

For immunofluorescence, sections were deparaffinized, rehydrated through a descending ethanol series to water, and washed in water, 0.2% Triton X‐100, and 1× PBS. Heat‐mediated antigen retrieval was performed in citrate buffer. Sections were blocked in 6% BSA, 5% FBS, 20 mm MgCl_2,_ and 0.2% Triton X‐100 in 1× PBS for 2 h at RT, then incubated overnight at 4 °C in a humidified chamber with the primary antibodies. As three of the four targets are detected by mouse antibodies, each primary antibody was applied to a separate tissue section: Anti‐Collagen IV [COL‐94] (Abcam, ab6311, RRID: AB_305414, 1:100), Anti‐Cytokeratin 10 [DE‐K10] (Abcam, ab9026, RRID: AB_2134912, 1:500), Anti‐Cytokeratin 14 [LL002] (Abcam, ab7800, RRID: AB_306091, 1:500), Anti‐Loricrin (Abcam, ab85679, RRID: AB_306950, 1:100), and Anti‐β‐tubulin III (Thermo Fisher Scientific, MA1‐118, RRID: AB_2536829, 1:1000) diluted in blocking solution. For localization in the same tissue sample, Anti‐Cytokeratin 10 (Abcam, ab76318, RRID: AB_1523465, 1:250) and Anti‐Cytokeratin 14 (Abcam, ab181595, RRID: AB_2811031, 1:250) were used together with the previously reported Anti‐β‐tubulin III. After washing in PBS, the mouse‐derived primary antibodies were detected with Goat anti‐Mouse IgG (H+L) Alexa Fluor 488 (Thermo Fisher Scientific, A‐11001, RRID: AB_2534069) and the rabbit‐derived primary antibody with Goat anti‐Rabbit IgG (H+L) Alexa Fluor 546 (Thermo Fisher Scientific, A‐11010, RRID: AB_2534077), at 1:500 in PBS. Nuclei were counterstained with DAPI (Thermo Fisher Scientific, cat# 62248, 1:10 000), and sections were imaged on a confocal Leica TCS SP5 II microscope.

### 4.5 FEM‐Based Analysis of Thermal Conduction

Simulations based on FEM were performed using COMSOL software (COMSOL Multiphysics v. 6.0. Stockholm, Sweden: COMSOL AB). 2D Axisymmetric geometry was used to build the model, with dimensions of the components obtained from direct measurements or manufacturers' data sheets. Culture medium was assumed to have similar thermal properties to water, while thermal properties of the IHSE components were determined from literature‐reported experiments [53]. Simulation was performed first in stationary conditions to evaluate the steady‐state temperature in the three domains of interest: upper epithelium, lower epithelium, and the bottom of the dermis. Then, a time‐varying simulation with a duration corresponding to that of the experiment was performed to simulate the change in temperature with time.

### 4.6 Waveform Analysis

Band‐passed filtered signals were processed using a MATLAB custom code. The signal was inverted to obtain positive peaks. The occurrence of peaks was determined using the following conditions: interspike intervals greater than 100 ms and amplitude greater than 25 µV. After determining the timestamp of the spike, the previous and following 30 samples were extracted. Parameters were determined as follows:

1. Amplitude was determined as the absolute value with respect to zero of the spike.
2. Thresholds were selected based on prior HD‐MEA studies and to minimize false‐positive spike detection (5.5 times the RMS of the signal value).
3. Trough‐Peak distance was measured as the prominence of the peak. Then the trough was determined as trough‐peak – amplitude.
4. Half‐peak width was automatically determined by MATLAB code, as indicated by the name.
5. Depolarization and repolarization slopes were determined as the tangent to the curve at the points closest to the half‐peak.

Interspike interval was then measured for each signal by comparing the difference between consecutive spikes to determine the mean value for the trace. These parameters were evaluated through statistical analysis, and the ones indicating the biggest significant differences were used to perform PCA. Samples were standardized after collating all the data together, and the components were evaluated to observe the presence of clusters. Centroids of the clusters were determined using the means of the distribution, then the Mahalanobis distance between the centroids was calculated using custom MATLAB code. Following this, the F‐statistic has been calculated according to the literature [54] and compared with the respective F‐critical to determine is distributions were significantly different.

Surrogate signals were generated through custom MATLAB code following the algorithm described for the Fourier Transform surrogates [40]. Briefly, from the original waveforms, Fast Fourier Transform (FFT) was computed. Then, a vector of random phases was generated and multiplied for the FFT vector of the signal. After finalizing the vector in order to make it complex conjugate symmetric, an inverse FFT was performed, resulting in the surrogate signal.

### 4.7 Statistical Analysis

All experiments were performed on biological triplicates. Mann‐Whitney statistical test was used to compare baseline values for the number of detected spikes and electrodes with stimulated ones, due to the values being dependent from each other. Pair Student *t*‐test was used for determining differences in fluorescence values and between wave‐analysis parameters. Pearson correlation was used to compare correlations between different waveforms for all types of stimulation. Mean and standard deviation for the Pearson's Correlation coefficient were evaluated by performing Fisher z‐transform on the coefficients, obtaining the Z‐Value of the Coefficient, evaluating mean and standard deviation, then applying the inverse transformation to obtain the values in the normal space. For statistical tests, a *P*‐value < 0.05 was considered significant. *p* < 0.05 is indicated with “^*^”, *p* < 0.01 with “^**^” and *p* < 0.001 with “^***^” in the figures. For Pearson's correlation, a value in the range of 0–0.25 was considered not correlated, 0.25–0.50 lowly correlated, 0.50–0.75 moderately correlated, and 0.75‐1 strongly correlated.

## Author Contributions

**Marika Sperduti**: methodology, investigation, visualization, Writing – original draft. **Paolo A. Netti**: conceptualization, supervision, writing – review and editing. **Costantino Casale**: conceptualization, methodology, investigation, writing – original draft. **Giorgia Imparato**: supervision, writing – review and editing. **Daniele Bellantoni**: conceptualization, methodology, investigation, software, data curation, visualization, writing – original draft. **Nevio L. Tagliamonte**: methodology, writing – original draft. **Loredana Zollo**: conceptualization, supervision, writing – review and editing.

## Funding

This work was partially supported by the EU Commission under the project SOMA (H2020‐ FETOPEN‐2019‐899822): Ultrasound peripheral interface and in vitro model of human somato‐sensory system and muscles for motor decoding and restoration of somatic sensations in amputees.

## Conflicts of Interest

The authors declare no conflicts of interest.

## Supporting information




**Supporting File 1**: advs77105‐sup‐0001‐SuppMat.docx.

**Supporting File 2**: advs77105‐sup‐0002‐VideoS1.zip.

## Data Availability

Data are available on reasonable request to the corresponding author.
