# Supplementary material for: An Innervated Human Skin Equivalent that Electrically Encodes Mechanical and Thermal Stimuli
Source: Adv Sci (Weinh). 2026 Aug 15:e77105. Online ahead of print. doi: 10.1002/advs.77105 (PMC13477244; doi:10.1002/advs.77105)
Supplement: Supplementary file 1 [file ADVS-9999-e77105-s002.docx]

**Supporting Information**

**S1:** **Generation and Evaluation of the Innervated Human Skin Equivalent integrated with HD-MEA systems.** Schematic representation of the Innervated Human Skin Equivalent Manufacturing Process. The procedure starts with the Human Dermal Equivalent (HDE) manufactured according to our previously published protocol (Imparato et al., 2013). Then, Schwann Cells are seeded, before seeding keratinocytes on top of the HDE+SC. With a combination of submerged and air-liquid interface culture conditions, the complete Human Skin Equivalent (HSE) is obtained, as demonstrated by histological sections and immunofluorescence. The following markers have been used: S-100 for Schwann Cells, Collagen IV for Dermal-epidermal junction, K14 and K10 for the epithelium and loricrine for the stratum corneum. In parallel with this process, Dorsal Root Ganglion (DRG) neurons have been cultured on high-density microelectrode array surface for 13 days. Then, by colturing the HSE with the active DRG for 6 days, we obtain the final Innervated Human Skin Equivalent model (IHSE).

**S2: Morphological focus on the innervation of the neuronal network at the interface with the HSE.** a) Time-lapse video showing neurites anchored to the dermal compartment of the HSE. Scale bar: 50 μm. Available as Video S2a b) 3D rotation video of the z-stack–based 3D reconstruction depicting the interface between the 2D neuronal network and the dermal compartment of the HSE: in gray, the newly synthesized collagen matrix visualized by second-harmonic generation (SHG) signal analysis; in green, the neurites identified by β3-tubulin immunostaining; and in light blue, the nuclei labeled with DAPI. Scale bar: 50 μm. Available as Video S2b c) 3D reconstruction of a paraffin-embedded section immunofluorescence; same color legend as in b. Note the presence of the tubulin marker near the dermal-epithelial junction. Scale bar: 100 μm. d) Immunofluorescence of histological section using anti-β-Tubulin III (green) and anti-Keratin 14 (red) with DAPI (light blue), with Collagen I identified using Second Harmonic Generation. This allows for co-localization between the fibroblasts stroma, basal layer of the epithelium and the innervating neurites e) 3D reconstruction of a z-stack showing the interactions between neurites and epithelial nuclei; same color legend as in b. Scale bar: 100 μm**.**

**S3: Histological section and immunofluorescence of neurites in the dermal compartment.** a) H&E staining of the IHSE, showing the dermis, epithelium, and stratum corneum. Scale bar: 500 μm. b–g) Immunofluorescence on paraffin-embedded sections labeled with β-tubulin III (green), Collagen I (gray), and DAPI (blue). Red arrows indicate neurites within the dermal compartment. The insets in b and f show magnified views of individual neurites (scale bars: 30 μm). All images confirm the presence of neurites within the dermal compartment, complementing those in Figure 2, which show neurites in the epithelium. Scale bars: 50 μm unless otherwise indicated.

**S4: spontaneous Electrophysiological outputs.** a) Macrograph of innervated HSE integrated with the HD-MEA system. Scalebar: 3 mm. b) Image obtained by aligning the corners of the Electrode View from the MaxLive software with the IHSE image acquired through a stereomicroscope. Red triangles indicate detected spikes on each electrode; a red box highlights a cluster of recording electrodes displaying synchronous activity across nearby neurons. Scale bar: 1 mm. c) Zoomed-in view at the center of a group of recording electrodes, showing the propagation of a spike from the central electrode recognizable by its highest amplitude toward the surrounding electrodes. (Timescale: 100 ms) d) Images obtained from the Axon Tracking assay at 9 days of innervation. The color map indicates the amplitude of the spike, which is higher at the center of the soma of the neurons, with the red lines corresponding to the detected axons. e) Table of the electrophysiological parameters measured through the Axon Tracking Assay at 9 days of innervation

**S5: long mechanical stimulation – evoked response.** a) Schematic of the experiment: three 100-μm indentations are applied to the surface of the IHSE, with 5 second of stimulation and 5 second intervals between stimulations. b) Example electrophysiological trace during mechanical stimulation: red line marks indentation onset, while green line indicates tip detachment. c) Overlaid detected spikes, aligning through the spike timestamp in to obtain an exemplary spike profile. d) Representative plot of detected spikes count (blue) overlaid with the tip position (red), illustrating correlation between mechanical force and firing rate increase. e) Representative raster plot aligned to indentation offset (red) and tip detachment (green), showing increase in number of active electrodes. Observed that data trace highlight the detach of skin equivalent from neural network in third touch.

**S6: mechanical Stimulation – Evoked Response (control validation).** a) Graphical representation of the experimental control where HSE were placed on HD-MEA surface without DRG neurons. b) Indication of the number of detected spikes during the recording, with evident absence in response. c) Graphical representation of the experimental control where the DRG network and the HSE were coculture for only 24 hours, resulting in a model lacking FNELS. d) Indication of the number of detected spikes during the recording, with evident response absence. e) Graphical representation of the experiment with Innervated HSE complete of FNELS. f) Indication of the number of detected spikes during the whole recording, where the evoked response to mechanical stimulation is noticeable. In all graph red line marks indicate tip contact, while green line indicates tip detachment.

**S7:** **FEM-based analysis of thermal conduction**. a) Macroscopic image of the setup, which has been used as starting point for the geometry of the section (Scalebar: 1 mm). b) Schematic representation of all the domains considered to reconstruct the geometry. c) Representation of the final geometry used for FEM software, after modelling all the domains established in b). d) Plot of the temperature simulation with the tip at 45°C. Red line corresponds to the top of the epithelium, green to the bottom of the epithelium/top of the dermis and blue to the bottom of the epithelium/neurons layer. From this simulation, we establish the value at the steady state and the different temperature at each layer as a function of time. Therefore, we can assume that the greatest temperature changes immediately following the stimulation happen in the epithelium layer, with slight changes in the bottom dermis. e) Plot of the temperature simulation with the tip at 10°C, with blue representing the bottom epithelium, green the bottom epithelium and red representing the top epithelium. Similarly to the other experiment, immediately following the stimulation, only the epithelium cools significantly, while the bottom of the dermis remains mostly at the same temperature.

**S8: Room Temperature-Evoked Electrical Activity of IHSE**. a) Schematic representation of the room temperature thermal stimulation experiment: the thermal tip at 28°C is put in contact with the IHSE b) Exemplary recorded signal from one of the electrodes during the evoked electrical activity: red dotted line indicates the moment of contact between the wet tip and the IHSE. c) Overlapping of all the detected spikes, aligning through the spike timestamp to obtain an exemplary spike profile. d) Exemplary plot of the recorded number of spikes, with the dotted line indicating the contact time between the tip and the IHSE. e) Exemplary raster plot, with the dotted line indicating the contact time between the tip and the IHSE. f) Quantitative analysis of the mean normalized number of spikes (n=3), where significant difference is not observed between spontaneous activity and stimulation at room temperature. g) Quantitative analysis of the mean normalized number of spikes (n=3), where no difference is observed between spontaneous activity and stimulation at room temperature).

**S9: Comparison among the three temperature ranges**. a) Quantitative analysis of the mean normalized number of spikes across the three temperature conditions (n=3), where significant differences are observed between warm and the other two conditions (P<0.001 in both cases), while differences between Cold and Room temperature are not significant. b) Quantitative analysis of the mean normalized number of active channels across the three temperature conditions (n=3), where significant differences are observed between warm and the other two conditions (P<0.001 between Warm and Cold, P<0.05 between Warm and Room), while differences between Cold and Room temperature are not significant.

**S10: Spatial and Temporal tracking of evoked activity**. Representative frames extracted from the Supplementary Video Thermal_Stimulation.mp4 showing the same IHSE sample immediately before and during thermal stimulation (a-b), and from Mechanical_Touch.mp4 showing the mechanical stimulation experiment (c-d). The background image and electrode layout remain unchanged because the recordings correspond to the same preparation. Red triangles indicate spikes detected by the recording system. The dynamic redistribution of spike activity is best appreciated in the accompanying Supplementary Videos. Scale bar = 500 μm.

**S11: *In-vivo* vs *in-vitro* Electrical Waverform Comparison**. A) Comparison between mean waveforms, with green for *in-vitro* and purple for *in-vivo.* Shaded areas correspond to the standard deviation of the average waveforms. Noticeable the difference in half-peak widths and spike amplitude between the two waves after standardization. Pearson’s correlation between the two waves results in a moderate correlation when considering all possible pairwise comparisons. B) Comparison between exemplary *in-vivo* waveform (purple) and surrogate waveform (orange). While power specter of the surrogate was equivalent to the original signals, the shape of the wave was distorted. Pearson’s coefficient indicates no correlation between surrogate signal and the *in-vivo* waveform. C) Distribution of the Pearson’s correlation coefficient for the *in-vitro/in-vivo* waveform comparison and the *in-vivo*/surrogate waveform comparison. The two distributions are significantly different (P<0.001).

**S12: Mechatronic platform.** a) mechatronic testbed integrated with recording unit of Maxwell Biosystems and accessory of videorecording unit and positioning and mechanical stimulator controller. b) mechanical stimulator with both IHSE integrated with HD-MEA relaxed and during compression by the tip of mechanical stimulator (scale bar = 1 mm) then Force and Displacement in the time graph of mechanical stimulation applied through 3 consecutive touches) thermal stimulator with both IHSE integrated with HD-MEA, in contact with the medium culture drop anchored to the thermal tip, with only drop on epidermal surface (scale bar = 3 mm).
